# Lack of Endogenous Adenosine Tonus on Sympathetic Neurotransmission in Spontaneously Hypertensive Rat Mesenteric Artery

**DOI:** 10.1371/journal.pone.0105540

**Published:** 2014-08-26

**Authors:** Joana Beatriz Sousa, Maria Sofia Vieira-Rocha, Carlos Sá, Fátima Ferreirinha, Paulo Correia-de-Sá, Paula Fresco, Carmen Diniz

**Affiliations:** 1 REQUIMTE/FARMA, Departamento de Ciências do Medicamento, Laboratório de Farmacologia, Faculdade de Farmácia, Universidade do Porto, Porto, Portugal; 2 MedInUP- Centro de Investigação Farmacológica e Inovação Medicamentosa, Universidade do Porto, Porto, Portugal; 3 Centro de Materiais, Universidade do Porto, Porto, Portugal; 4 Laboratório de Farmacologia e Neurobiologia/UMIB, Instituto de Ciências Biomédicas Abel Salazar, Universidade do Porto, Porto, Portugal; Indiana University School of Medicine, United States of America

## Abstract

**Background:**

Increased sympathetic activity has been implicated in hypertension. Adenosine has been shown to play a role in blood flow regulation. In the present study, the endogenous adenosine neuromodulatory role, in mesenteric arteries from normotensive and spontaneously hypertensive rats, was investigated.

**Methods and Results:**

The role of endogenous adenosine in sympathetic neurotransmission was studied using electrically-evoked [^3^H]-noradrenaline release experiments. Purine content was determined by HPLC with fluorescence detection. Localization of adenosine A_1_ or A_2A_ receptors in adventitia of mesenteric arteries was investigated by Laser Scanning Confocal Microscopy. Results indicate a higher electrically-evoked noradrenaline release from hypertensive mesenteric arteries. The tonic inhibitory modulation of noradrenaline release is mediated by adenosine A_1_ receptors and is lacking in arteries from hypertensive animals, despite their purine levels being higher comparatively to those determined in normotensive ones. Tonic facilitatory adenosine A_2A_ receptor-mediated effects were absent in arteries from both strains. Immunohistochemistry revealed an adenosine A_1_ receptors redistribution from sympathetic fibers to Schwann cells, in adventitia of hypertensive mesenteric arteries which can explain, at least in part, the absence of effects observed for these receptors.

**Conclusion:**

Data highlight the role of purines in hypertension revealing that an increase in sympathetic activity in hypertensive arteries is occurring due to a higher noradrenaline/ATP release from sympathetic nerves and the loss of endogenous adenosine inhibitory tonus. The observed nerve-to-glial redistribution of inhibitory adenosine A_1_ receptors in hypertensive arteries may explain the latter effect.

## Introduction

Increased sympathetic activity has been implicated in the pathophysiology of hypertension since it drives to an enhancement of vasoconstriction.[1], [2] Vascular sympathetic activity can be regulated by several endogenous substances, such as adenosine.

Extracellular adenosine can either be released as such, *via* nucleoside transporters, or produced from extracellular catabolism of released adenine nucleotides, namely ATP, from distinct cells including neurons. ATP is then sequentially dephosphorylated into ADP, AMP and adenosine. [3] Besides its action at the synapse, adenosine may function as a non-synaptic signalling molecule upon diffusion from its local of origin influencing neurotransmission, inflammation and immune responses.[4] Adenosine effects occur through activation of four G-protein coupled receptors, adenosine A_1_, A_2A_, A_2B_ and A_3_ receptors.[5]

In vessels, the involvement of adenosine receptors in sympathetic modulation has been described both in arteries[6]–[13] and in veins.[12] A reduced effect mediated by selective adenosine A_1_, but not A_2A_ receptor agonists in sympathetic vascular neurotransmission in hypertensive state has been reported.[13] Nevertheless, the endogenous adenosine role in vascular sympathetic neurotransmission remains to be clarified, particularly whether the endogenous adenosine levels may have a pathophysiological impact in hypertension.

We postulate that the effects of endogenously generated adenosine are also impaired in hypertensive individuals leading to increased vascular sympathetic activity. The study was undertaken in mesenteric arteries from normotensive (Wistar-Kyoto, WKY) and spontaneously hypertensive rats (SHR), a well-establish model of hypertension,[14], [15] to determine whether endogenous adenosine has a role in the modulation of sympathetic activity and if this role is preserved in hypertensive individuals. Moreover, the regional distribution/localization and relative amount of adenosine receptors (A_1_ and A_2A_ subtypes) in the two animal strains was also evaluated.

## Materials and Methods

### Animals

Adult male WKY and SHR (12 weeks old, 270–350 g; Charles River, Barcelona, Spain) were used. Handling and care of animals were conducted according to the European guidelines (Directive 2010/63/EU) on the protection of animals used for scientific purposes in agreement with the NIH guidelines. This study was carried out in strict accordance with the recommendations in the Guide for the Care and Use of Laboratory Animals of the National Institutes of Health. The protocol was approved by the Committee on the Ethics of Animal Experiments of the University of Porto (Permit Number: 13/11/2013). Animals were sacrificed using guillotine. Two animals per experiment were used and from each mesenteric artery four segments (4–7 mg) were obtained. From each animal, no more than two tissue preparations were submitted to identical treatments.

### Chemicals

The following drugs were used: levo-[ring-2,5,6-3H]-noradrenaline, specific activity 41.3 Ci/mmol, was from DuPont NEN (I.L.C., Lisboa, Portugal); desipramine hydrochloride, 8-cyclopentyl-1,3-dipropylxanthine (DPCPX), 7-(2-phenylethyl)-5-amino-2-(2-furyl)-pyrazolo-[4,3-e]-1,2,4-triazolo[1,5-c] pyrimidine (SCH 58261), S-(4-Nitrobenzyl)-6-thioinosine (NBTI) and 5-Iodotubericidin (ITU), (8R)-3-(2-Deoxy-β-D-erythro-pentofuranosyl)-3,4,7,8-tetrahydroimidazo[4,5-d][1], [3]diazepin-8-ol (pentostatin), α,β-methylene ADP, N6-cyclopentyladenosine (CPA), 2-p–(2-carboxyethyl)phenethylamino-5′-N-ethylcarboxamidoadenosine hydrochloride (CGS 21680) were purchased from Sigma-Aldrich (Sintra, Portugal). The following antibodies were used: rabbit polyclonal anti-A1 (epitope corresponding to amino acids 287-326 mapping at the C-terminus of human adenosine A_1_ receptors; sc-28995), anti-A_2A_ (epitope corresponding to amino acids 331-412 mapping at the C-terminus of human adenosine A_2A_ receptors; sc-13937) were purchased from Santa Cruz Biotechnology, Inc., CA, USA; mouse monoclonal anti-tyrosine hydroxilase antibody (TH(45): sc-136100, Santa Cruz Biotechnology, Inc., CA, USA and MAB318, Millipore Corporation, CA, USA); anti-glial fribillary acidic protein (GFAP) mouse monoclonal antibody (G6171, Sigma-Aldrich, Inc., USA) and rabbit GFAP polyclonal antibody (18-0063, Invitrogen, Life Technologies, SA, Madrid, Spain). The following fluorescent probes were used: Alexa Fluor 488 goat anti-mouse IgG (H+L) antibody, highly cross-adsorbed and Alexa Fluor 647 goat anti-rabbit IgG (H+L) antibody, highly cross-adsorbed (Molecular Probes) secondary fluorescent antibodies (Invitrogen, Life Technologies, SA, Madrid, Spain); vectashield mounting medium with DAPI (Vector Laboratories, UK). Stock solutions were made up in dimethylsulphoxide (DMSO: 0.01% v/v, final concentration) or ultrapure water and diluted in superfusion medium immediately before use. DMSO was added to the superfusion medium (final concentration 0.01%), in parallel control experiments.

### [^3^H]-Noradrenaline release experiments

Evaluation of [^3^H]-noradrenaline release experiments was carried out as previously described.[9]–[13] Arteries were pre-incubated in 2 mL Krebs-Henseleit solution containing 0.1 µmol/L [^3^H]-noradrenaline (for 60 min at 37°C) and transferred into superfusion chambers, superfused with [^3^H]-noradrenaline-free medium (1 mL/min; constant rate: Krebs-Henseleit solution with desipramine 400 nmol/L to inhibit noradrenaline's neuronal uptake). Two periods of electrical stimulation (5 Hz, 100 pulses, 1 ms, 50 mA; Hugo Sachs Elektronik, March-Hugstetten, Germany) were applied, S_1_ and S_2_, with 30 min intervals (t = 90 min and t = 120 min, respectively). The superfusate was collected each 5 min period from 85 min of superfusion onwards. At the end of the experiments (t = 130 min), tritium was measured in superfusate samples and solubilized arteries (sonicated 1 h with 2.5 mL perchloric acid (0.2 mol/L)) by liquid scintillation spectrometry (LS 6500, Beckman Instruments, Fullerton, USA) after adding 6 mL of a scintillation mixture (OptiPhase ‘Hisafe’ 3, PerkinElmer, I.L.C., Lisboa, Portugal) to each sample.

Tissue labelling with [^3^H]-noradrenaline and the evaluation of electrically-evoked tritium overflow changes were performed as previously described.[12], [13] Effects of agonists (CPA, CGS 21680), of antagonists (DPCPX, SCH 58261), and of enzyme (pentostatin, ITU, α,β-methylene ADP) and nucleoside transport (NBTI) inhibitors were studied.

### Laser scanning confocal microscopy (LSCM) experiments

Immunohistochemistry procedures were previously described.[13] Briefly, four tissue preparations were obtained from each artery and immediately placed in cold phosphate buffer solution (PBS; in g/L): NaCl 8.0, Na_2_HPO_4_.2H_2_O 0.77, KCl 0.20, KH_2_PO_4_ 0.19 (pH 7.2). Each preparation was longitudinally opened and fixed (paraformaldehyde 4% PBS; 50 min; room temperature). After two 15 min PBS washing cycles, artery segments were incubated with primary antibodies raised against rabbit polyclonal individual adenosine receptor subtypes (anti-A_1_ or anti-A_2A_, 1:200 dilution, overnight, 4°C) and mouse monoclonal anti-tyrosine hydroxilase (TH, 1:10 dilution, overnight, 4°C) or mouse monoclonal anti-glial fribrilary acidic protein (GFAP, 1:200 dilution, overnight, 4°C). Thereafter, tissues were incubated with Alexa 647 anti-rabbit and Alexa 488 anti-mouse fluorescent secondary antibodies (1:1000 dilution, 1 h, room temperature). Negative controls were performed by omitting primary antibodies. After two PBS washing cycles, tissue preparations were mounted with antifading agent (vectashield mounting medium with DAPI, Vector Laboratories, UK), with the adventitial side facing up.

Preparations were visualized with Olympus FluoView FV1000 fluorescence confocal microscope system with a x60 oil immersion lens. Stacks of 1 µm thick serial optical images were captured from the entire adventitial layer, which was identified by the shape and orientation of the nuclei. Image acquisition was performed always under the same laser power, brightness and contrast conditions. Adventitia was scanned along each mesenteric artery and the resulting images were reconstructed separately for each wavelength.

### ε-derivatives assay in artery superfusates

1,N^6^-Etheno (ε)-modified purines have been previously used for purine quantification in tissue superfusates. [3], [16]–[19] Briefly, mesenteric artery segments were superfused (Krebs-Henseleit, 1 mL.min^−1^) and electrically stimulated twice (S_1_-S_2_; 5 Hz, 100 pulses, 1 ms, 50 mA) 30-min apart ( t = 90 min and t = 120 min). 5-min superfusates were collected and heated at 80°C. From the collected samples, 910 µL were incubated with 90 µL of chloroacetaldehyde for 50 min at 70°C in a dry bath incubator (Heraeus Instruments, Hanau, Germany). Reactions were stopped by placing samples on ice. Identification of the ε-derivatives (ε-ATP, ε-ADP, ε-AMP and ε-adenosine) formed in these collected samples was confirmed by HPLC using a fluorescent detector (model LS20; Perkin Elmer, Beconsfield, UK) at 230 nm excitation and 420 nm emission wavelengths. The stationary phase was 5 µm particle size packed in a 250 cm long by 4 mm internal diameter ODS- (C18) column and matching 1 cm long by 3.3 mm diameter direct-connect guard column (ACE-Advanced Chromatography technology, Aberdeen, Scotland) in a gradient HPLC system (306 and 811C Gilson, Gilson Medical Electronics, Middleton, WI, USA). The column was kept at room temperature (20-22°C). The mobile phase consisted of a solution of 87 mmol/L KH_2_PO_4_ and 10.6 mmol/L Na_2_HPO_4_ (pH 6.0) as buffer A; buffer B was made up 25% methanol and 75% buffer A. Gradient elution was used according to the following linear program: from 0 to 20 min of elution, a convex gradient from 0 % to 100% of Buffer B at a flow rate of 1 mL/min; from 0 to 3 min of elution, an increase from 0% to 25% of buffer B at a flow rate of 1 mL/min; from 3 to 7 min of elution, 25% of buffer B at a flow rate of 1 mL/min; and from 8 to 12 min of elution an increase from 25% to 100% of buffer B at a flow rate of 1 mL/min; from 12 to 20 min of elution, 100% of buffer B at a flow rate of 1 mL/min. The run time of 20 min and the post-run time was 5 min.

### Data Analysis

#### Measurement of drug effects on electrically-evoked tritium overflow

Electrically-evoked tritium overflow from artery segments incubated with [^3^H]-noradrenaline has been shown to reflect action potential-evoked neuronal noradrenaline release and drug-induced changes in evoked tritium overflow are assumed to reflect changes in neuronal noradrenaline release. Effects of drugs added after S_1_ on electrically-evoked tritium overflow were evaluated as ratios of the overflow elicited by S_2_ and the overflow elicited by S_1_ (S_2_/S_1_). S_2_/S_1_ ratios obtained in individual experiments in which a test compound A was added after S_1_ were calculated as a percentage of the respective mean ratio in the appropriate control group (solvent instead of A). When the interaction of A, added after S_1_, and a drug B added 5 min before S_2_, was studied, the “appropriate control” was a group in which A alone was used.

#### Laser Scanning Confocal Microscopy images quantification

Quantitative analysis of confocal z-stacks images was performed using image analysis software (PAQI, CEMUP, Porto, Portugal). Briefly, a sequential routine was designed and developed to analyse each fluorescent signal used. PAQI software measured the surface area and strength of the fluorescence signal marking the postganglionic nerves, the surface area and strength of the fluorescence signal marking the receptors and determined the surface area of attachment of the receptors on the nerves as well as the intensity of fluorescence of the receptors on nerves (corrected for background).

#### Quantification of the ε-derivatives formed in collected artery superfusates

The amount of adenine nucleotides and adenosine (pmol/mg of tissue) in each collected sample was estimated from calibration curves of purine standards (derivatized as described above for the samples), run with every set of 20 samples.

#### Statistics

Results are expressed as mean±s.e.m. and *n* denotes the number of tissue segments. Differences of means were compared for significance using one- or two-way ANOVA, followed by *post-hoc* Holm-Sidak's multicomparison *t* test. A P value lower than 0.05 was considered to denote statistically significant differences.

## Results

### Noradrenaline release from mesenteric sympathetic nerve terminals

Stimulation (100 pulses/5 Hz) significantly increased tritium outflow from mesenteric artery of both WKY and SHR. In the absence of drugs other than desipramine (400 nmol/L), the average basal overflow (b_1_, fractional release), immediately before S_1_, was similar in WKY (0.070±0.008; n = 15) and SHR (0.071±0.003; n = 11) mesenteric arteries, whereas electrically-evoked tritium overflow (S_1_, ratio of the total tritium content of the tissue) was more pronounced in SHR mesenteric arteries (0.358±0.05; n = 11; p<0.05) comparatively to those from WKY (0.229±0.037; n = 15). In control conditions, basal outflow and electrically-evoked tritium overflow remained constant throughout experiments, with b_n_/b_1_ and S_n_/S_1_ average values close to unity (data not shown).

### Role of endogenous adenosine in vascular sympathetic neurotransmission

**Figure 1:**
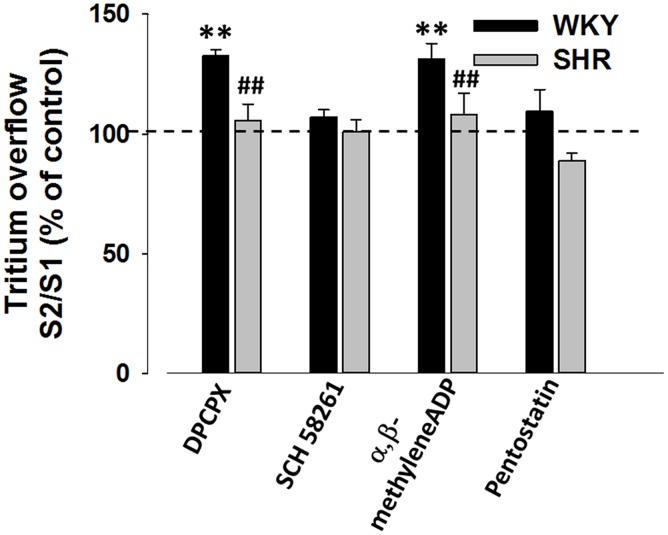
Influence of endogenous adenosine on electrically-evoked tritium overflow from WKY and SHR mesenteric arteries: interaction with DPCPX, SCH 58261, α,β-methylene ADP or pentostatin. Tissues were electrically stimulated (S_1_-S_2_: 100 pulses, 5 Hz). Drugs were added after S_1_ and kept until the end of the experiment. Values are mean±s.e.m. from 4–12 segments. Significant differences from the appropriate control: *P<0.05; from WKY: ^##^ P<0.001.

Endogenous adenosine-mediated effects in mesenteric artery sympathetic neurotransmission were evaluated by blocking the high affinity adenosine receptor subtypes (A_1_ and A_2A_) with the selective antagonists, DPCPX and SCH 58261, respectively. In the presence of DPCPX (100 nmol/L), a facilitation of electrically-evoked tritium overflow was observed (Figure 1). This finding is compatible with the occurrence of a tonic inhibition mediated by endogenous adenosine *via* adenosine A_1_ receptor activation. However, this effect was only observed in WKY mesenteric arteries. SCH 58261 (20 nmol/L) did not modify tritium overflow in mesenteric arteries from both strains, discarding the occurrence of a putative tonic adenosine A_2A_ receptor-mediated facilitation. Upon reducing endogenous adenosine levels by preventing AMP (formed from dephosphorylation of ATP released after nerve stimulation) conversion into adenosine, through inhibition of ecto-5′-nucleotidase (with α,β-methylene ADP, 10 µmol/L), we observed a facilitation of similar magnitude of that detected in the absence of tonic adenosine A_1_ receptor inhibition (revealed by DPCPX treatment). Likewise, this effect was only observed in WKY mesenteric arteries.

**Figure 2:**
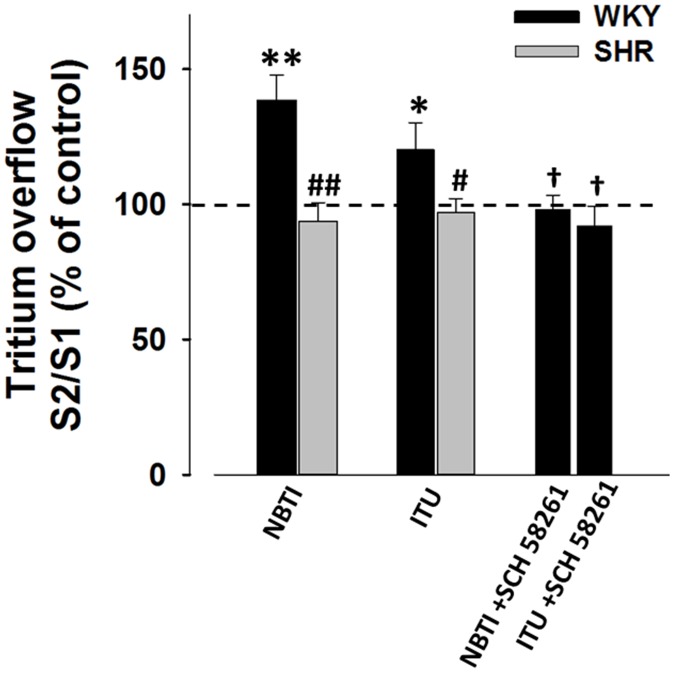
Effects of NBTI or ITU in the absence or presence of SCH 58261 on the electrically-evoked tritium overflow from WKY and SHR mesenteric arteries. Tissues were electrically stimulated (S_1_-S_2_: 100 pulses, 5 Hz). Drugs were added after S_1_ and kept until the end of the experiment. Values are mean±s.e.m. from 4-12 segments. Significant differences from the appropriate control: * P<0.05, * P<0.05; from WKY: ^##^ P<0.001; from the inhibitor alone: † P<0.05.

Pentostatin (10 µmol/L), an adenosine deaminase inhibitor, did not alter the electrically-evoked tritium overflow (Figure 2). However, NBTI (5 µmol/L), a bidirectional equilibrative nucleoside transporter inhibitor and ITU (100 nmol/L), an adenosine kinase inhibitor were able to increase tritium overflow, but only in WKY mesenteric arteries (Figure 2). Moreover, the facilitatory effects observed in the presence of ITU or NBTI were completely antagonised by SCH 58261 (Figure 2). In contrast, electrically-evoked tritium overflow from SHR mesenteric arteries was unaffected by pharmacological manipulation of endogenous adenosine levels (Figures 1 and 2).

### ATP and adenosine levels in WKY and SHR mesenteric arteries

**Figure 3:**
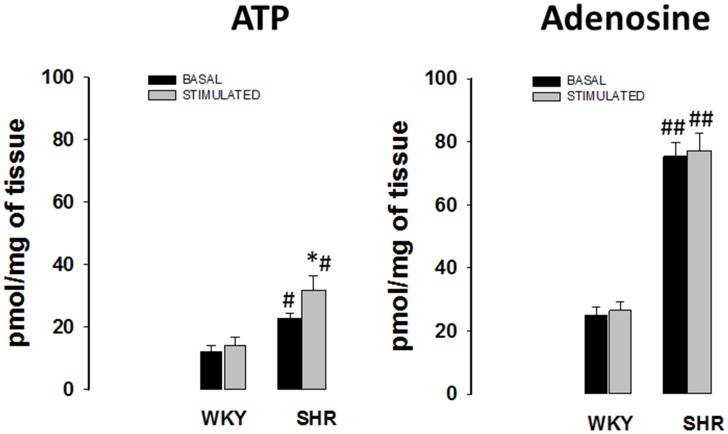
Basal and electrically-evoked ATP (A) and adenosine (B) release in mesenteric arteries from WKY and SHR. Tissues were electrically stimulated (S_1_-S_2_: 100 pulses, 5 Hz). Significant differences from basal conditions: *P<0.05; from WKY: ^#^ P<0.05; ##P<0.001.

Noradrenaline/ATP co-transmission has been demonstrated to occur in mesenteric artery.[20], [21] Endogenous levels of ATP and adenosine were significantly higher in superfusates from SHR mesenteric arteries, comparatively to those of WKY (Figure 3), both under basal conditions and after electrical stimulation (5 Hz/100 pulses). Only minute amounts of ADP and AMP were found (data not shown). Moreover, stimulation increased ATP released amounts comparatively to the amounts observed in basal conditions, but only in SHR tissues. Surprisingly, adenosine levels, before and after stimulation, were similar in arteries from both strains, although still higher than those of ATP.

### Effects of adenosine A_1_ and A_2A_ receptor agonists on vascular sympathetic neurotransmission

CPA (a selective adenosine A_1_ receptor agonist, 100 nmol/L) inhibited electrically-evoked tritium overflow in mesenteric arteries from both strains. This inhibition was more pronounced in WKY (63.28±3.9%; n = 6; p<0.05) than in SHR (74.70±2.86%; n = 14; p<0.05). Conversely, the selective adenosine A_2A_ receptor agonist, CGS 21680 (100 nmol/L), facilitated tritium overflow to similar extent: 123.64±5.18% (n = 4; p<0.05) and 121.62±7.73% (n = 4; p<0.05) in WKY and SHR mesenteric arteries, respectively. Effects elicited by adenosine receptor agonists were supressed by pre-incubation with the corresponding selective adenosine receptor antagonists, DPCPX (100 nM) and SCH 58261 (20 nM). These results demonstrate that mesenteric arteries from WKY and SHR exhibit functional adenosine A_1_ and A_2A_ receptors, which can be selectively activated by stable adenosine analogues. These results suggest that activation of adenosine A_2A_ receptors is largely preserved in hypertensive rats but the adenosine A_1_ receptor-mediated inhibition is somehow compromised in these animals.

### Localization of adenosine A_1_ and A_2A_ receptors in vascular sympathetic neurons in the adventitia of WKY and SHR mesenteric arteries

**Figure 4:**
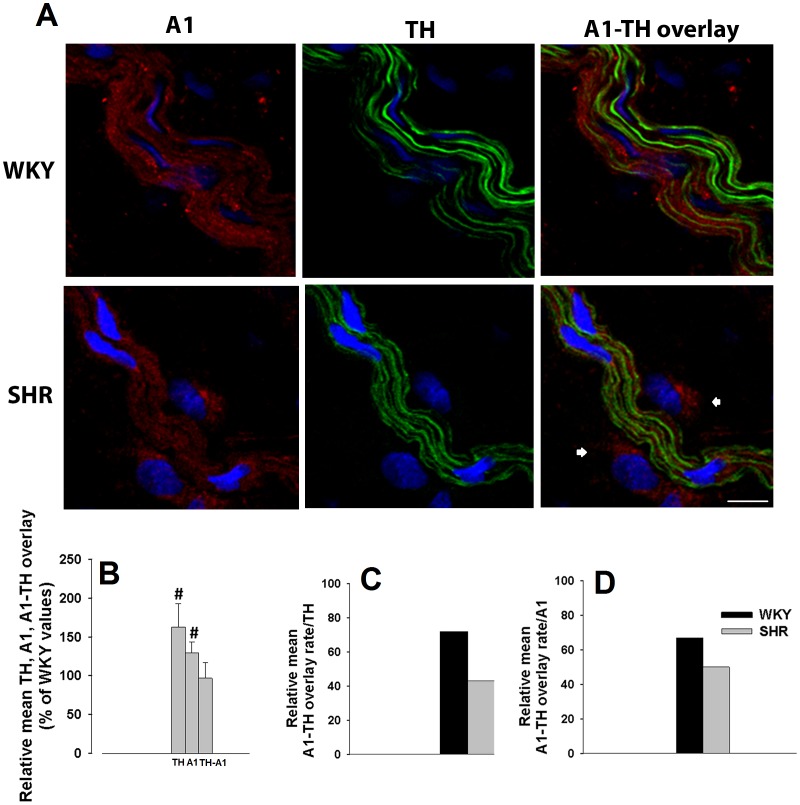
Laser scanning confocal microscopy representative images of WKY and SHR mesenteric arteries exhibiting (A) adenosine A_1_ receptor (red), TH (green) and overlay of A_1_-TH immunoreactivities, nuclei (blue); (B) Relative means of TH, A_1_ and TH-A_1_ overlay expressed as percentage of WKY values. (C) Mean percentage of overlay rate with TH (D) and mean percentage of overlay rate with A_1_ are depicted. Arrows highlight non-neuronal cells. Images are reconstructions from 9–28 serial optical sections analyzed using PAQI software. Values are mean±s.e.m.; 3–4 animals. Significant differences from WKY: ^#^ P<0.05, ^##^ P<0.001. Scale bar  =  20 µm.

In WKY and SHR mesenteric arteries, adenosine A_1_ receptor immunoreactivity showed considerable overlay, but did not co-localize, with the sympathetic neuronal marker (TH; Figure 4A), indicating that they might be localized on the same cellular structure (postganglionic sympathetic nerves). This agrees with the fact that adenosine A_1_ receptors are membrane receptors while tyrosine hydroxylase (TH) is an enzyme localized inside sympathetic neurotransmitter storage vesicles.[22] Adenosine A_1_ receptor immunoreactivity in non-neuronal cells was also observed, particularly in mesenteric arteries from SHR. TH and A_1_ immunoreactivities in SHR exceeds those observed in WKY arteries (Figure 4B). However, overlaid TH and A_1_ immunoreactivities were roughly similar in arteries from both strains (Figure 4B). Figure 4C shows that adenosine A_1_ receptor and TH overlaid immunoreactivities are markedly reduced in SHR comparatively to WKY tissues, when data was normalized by total TH immunoreactivity: 70% of sympathetic neurons exhibit adenosine A_1_ receptor immunoreactivity in WKY *versus* only 40% observed in SHR. Adenosine A_1_ receptor immunoreactivity was also observed in cells other than sympathetic neurons: 33% in WKY and 50% in SHR mesenteric arteries (Figure 4A and 4D).

**Figure 5:**
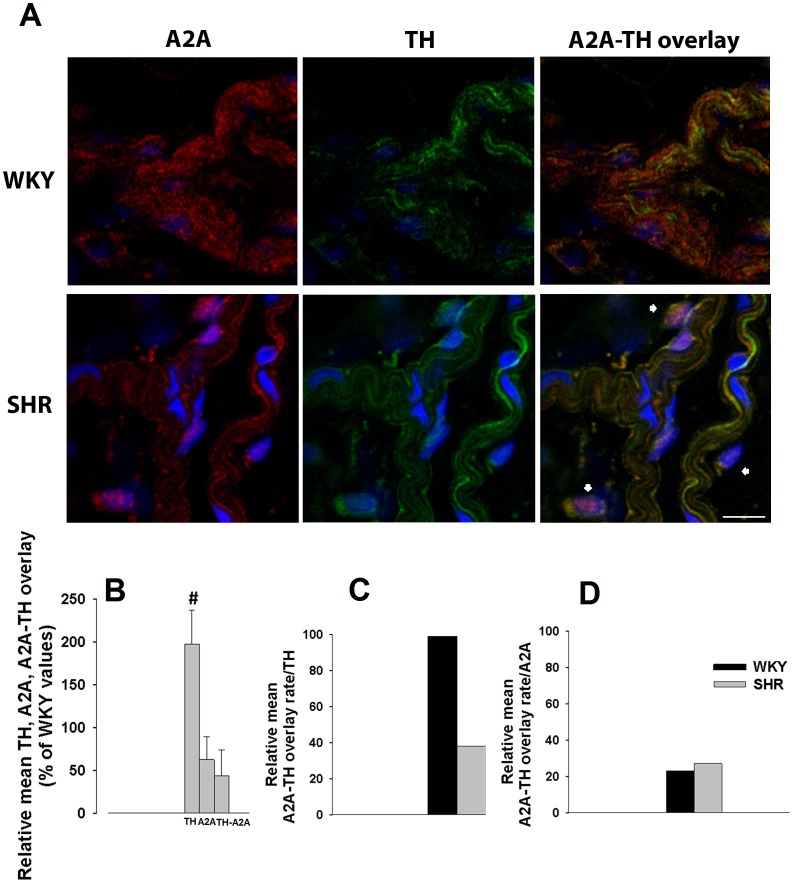
Laser scanning confocal microscopy representative images of WKY and SHR mesenteric arteries exhibiting (A) adenosine A_2A_ receptor (red), TH (green) and overlay of A_2A_-TH immunoreactivities, nuclei (blue); (B) Relative means of TH, A_2A_ and TH-A_2A_ overlay expressed as percentage of WKY values. (C) Mean percentage of overlay rate with TH (D) and mean percentage of overlay rate with A_2A_ are depicted. Arrows highlight non-neuronal cells. Images are reconstructions from 11–20 serial optical sections analyzed using PAQI software. Values are mean±s.e.m.; 3–4 animals. Significant differences from WKY: ^#^P<0.05, ^##^ P<0.001. Scale bar  =  20 µm.

Similar analysis of adenosine A_2A_ receptor and tyrosine hydroxylase (TH) was performed in WKY and SHR mesenteric arteries (Figure 5). TH immunoreactivity in SHR exceeds that observed in WKY (Figure 5B), while A_2A_-TH overlaid immunoreactivities are lower in SHR arteries. Figure 5C shows that A_2A_-TH, normalized by total amount TH immunoreactivity, is significantly reduced in SHR (38%) comparatively to WKY tissues, where almost all sympathetic neurons (99% of TH-positive immunoreactivity) also exhibit adenosine A_2A_ receptor immunoreactivity. Moreover, the percentage of cells exhibiting adenosine A_2A_ receptor immunoreactivity other than sympathetic neurons was similar in WKY (77%) and SHR arteries (73%) (Figure 5D).

### Localization of adenosine A_1_ and A_2A_ receptors in Schwann cells of the adventitia of WKY and SHR mesenteric arteries

**Figure 6:**
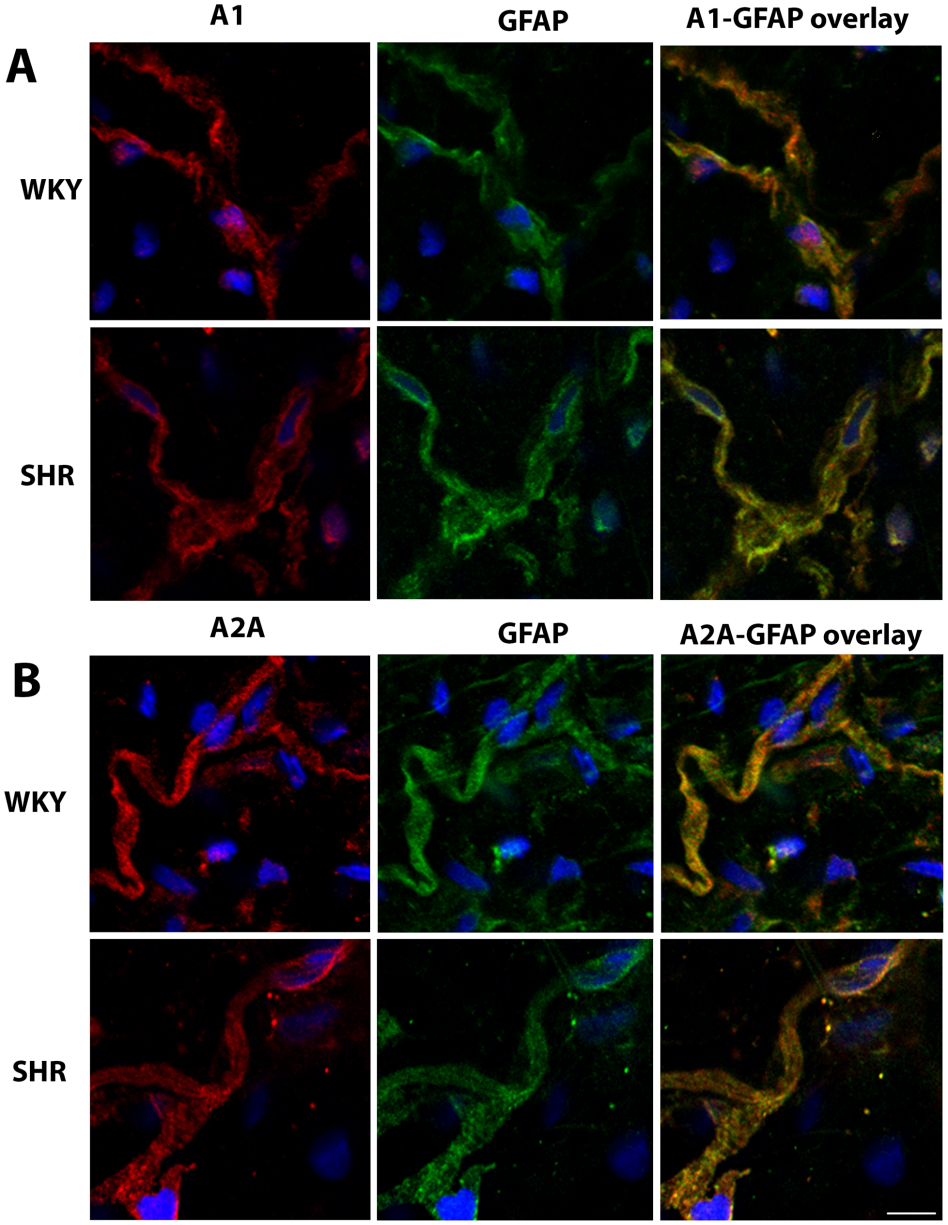
Laser scanning confocal microscopy images of adenosine A_1_ (panel A) and A_2A_ receptor (panel B) immunoreactivities in GFAP-immunoreactive (Schwann) cells localized in adventitia from WKY and SHR mesenteric arteries; 3–4 animals. Scale bar  =  20 µm.

In the adventitia of mesenteric artery several cell types can co-exist.[23], [24] Since we have observed adenosine A_1_ (Figure 4D) and A_2A_ receptor (Figure 5D) immunoreactivities outside sympathetic neurons and since these neurons are wrapped with Schwann cells, we hypothesized that the non-neuronal cells exhibiting adenosine A_1_ or A_2A_ receptor immunoreactivities were Schwann cells. GFAP (peripheral glial cell marker)[25] immunoreactivity overlaid with adenosine A_1_ (Figure 6A) or A_2A_ (Figure 6B) receptor immunoreactivities in the adventitia of both strains. Therefore, in addition to sympathetic nerves, Schwann cells also present adenosine A_1_ and A_2A_ receptors.

## Discussion

There is a gap in the knowledge regarding the complex interplay between receptor expression and the role of the endogenous neuromodulator, adenosine, on vascular sympathetic neurons. This study shows that endogenous adenosine contributes to down-modulate noradrenaline release from sympathetic neurons through activation of adenosine A_1_ receptors in normotensive mesenteric arteries, but this effect is completely lost in hypertensive arteries justifying the observed increase of noradrenaline release.

In normotensive mesenteric arteries removal of the inhibitory tone of adenosine either by inhibiting ecto-5′-nucleotidase or by selectively blocking adenosine A_1_ receptors caused a facilitation of noradrenaline release from stimulated sympathetic neurons up to 32%. Therefore, it seems that, under physiological conditions, endogenous adenosine contributes to restrain transmitter release from stimulated mesenteric arteries *via* activation of inhibitory adenosine A_1_ receptors, as previously demonstrated.[26] Data also indicate that the adenosine A_1_ receptor inhibitory tonus is mediated predominantly by adenosine originated from metabolism of released adenine nucleotides. These results along with the observation that the amount of adenosine in superfusates was unaffected upon stimulation were rather surprising. Nevertheless, our findings gain physiological significance if one considers that inhibitory adenosine A_1_ receptors operate to restrain noradrenaline release under basal conditions, as a consequence of adenosine accumulation from hydrolysis of ATP released from neighbouring cells. This scenario may change towards putative activation of P2 receptors, along with adenosine receptors, in hypertensive arteries[2] a possibility if one takes into consideration the increased ATP levels found in stimulated SHR superfusates.

Transmitters release modulation ascribed to extracellular adenosine accumulation depends both on its formation and on cellular uptake and deamination.[27]–[29] We showed that adenosine deaminase inhibition failed to modify noradrenaline release, causing minor changes in extracellular adenosine accumulation. Interestingly, NBTI was able to increase noradrenaline release, suggesting that adenosine uptake may be the dominant adenosine inactivation pathway in mesenteric arteries. NBTI enhancement of noradrenaline release was antagonised by SCH 58261, indicating that this effect is mediated by adenosine A_2A_ receptors, activated by endogenous adenosine (Figure 2). These apparent contradictory results, showing that SCH 58261 was unable to modify transmitter release from stimulated sympathetic neurons (Figure 1), may be explained due to differences in *K_d_* ascribed to inhibitory adenosine A_1_ and facilitatory A_2A_ receptors, the latter requiring concentrations two-fold higher than those required to activate adenosine A_1_ receptors.[30] Nucleoside transporter inhibition seems to increase extracellular adenosine to levels high enough to activate adenosine A_2A_ receptors, while in its absence the amount of adenosine may be insufficient to activate these receptors. Therefore, in conditions that favour extracellular adenosine accumulation, this nucleoside may reach concentrations higher enough to activate adenosine A_2A_ receptors, as described in other tissues.[27]–[29]

To our knowledge, this is the first study demonstrating that the endogenous adenosine neuromodulatory role of sympathetic transmission is significantly impaired in mesenteric arteries from hypertensive rats. In SHR mesenteric arteries we failed to detect both adenosine A_1_ and A_2A_ receptor mediated effects (after NBTI-induced extracellular adenosine accumulation). This is occurring despite the extracellular levels of both ATP and adenosine were significantly higher in hypertensive mesenteric arteries than those measured in normotensive artery superfusates. These differences on adenosine neuromodulation may be explained by changes in adenosine A_1_ and/or A_2A_ receptors: i) activity/desensitization, ii) downregulation or iii) redistribution into other organelle or cells. In this regard, we showed, using enzymatically stable and selective adenosine receptor agonists, that neuromodulatory activity of adenosine A_1_ but not of A_2A_ receptors is significantly impaired in hypertensive mesenteric arteries.

Laser scanning confocal microscopy data confirmed previous reports of a sympathetic hyperinnervation in hypertensive mesenteric arteries.[31]–[34] Interestingly, increases in the number and thickness of sympathetic nerve fibres observed in SHR mesenteric arteries was not accompanied by a correspondent enhancement of adenosine A_1_ and/or A_2A_ receptors overlaying these neurons: a decrease of both adenosine A_1_ (from 70% to 40%) and A_2A_ receptors (from 99% to 40%) in sympathetic neurons in hypertensive *versus* normotensive mesenteric arteries was observed. These lower amounts can explain, at least in part, the lack of adenosine inhibitory (and facilitatory) tone regulating noradrenaline release from stimulated SHR mesenteric arteries, leading to higher extracellular noradrenaline levels.

Data also show, for the first time, that 33% of adenosine A_1_ receptors and 77% of adenosine A_2A_ receptors, in WKY mesenteric artery adventitia, are present in other cells than sympathetic neurons (TH-immunoreactive cells). While the amount of adenosine A_1_ receptors localized in these cells (Schwann cells) increased to roughly 50% in SHR arteries, we observed no changes in the amount of adenosine A_2A_ receptors in arteries from both strains. This finding might explain the small (∼20%) relative increase in the amount of adenosine A_1_ receptors in hypertensive mesenteric arteries, suggesting a neuron-to-glia redistribution of adenosine A_1_ receptors in hypertensive arteries. To our knowledge, this is also the first time where distribution/redistribution of adenosine A_1_ or A_2A_ receptors between neurons and Schwann cells in hypertensive arteries has been reported. Changes in the localization of adenosine receptors and the increased amount of extracellular adenosine observed in SHR mesenteric arteries suggest that receptor desensitization may be the main reason for adenosine receptor activity impairment observed in hypertensive arteries. Indeed, the lack of tonic adenosine A_1_ receptor-mediated inhibition in these arteries may contribute to increase noradrenaline release from stimulated sympathetic nerves. This provides a convincing explanation for the dominance of noradrenaline as neurotransmitter in hypertensive rats.[35] Accordingly, decreases in adenosine A_1_ receptor-mediated inhibitory tonus may cause a profound impact in vascular reactivity, contributing to hypertension. Moreover, the amount of ATP released from sympathetic nerves was higher in SHR than in WKY mesenteric arteries which correlates well with a previous work where an enhanced purinergic function was described, revealing ATP as a vasoconstrictor in SHR arteries.[2]

## Conclusions

Results from this work highlight the role of purines in hypertension. Data showed that the increase in sympathetic activity in hypertensive arteries may be partially due to an higher ATP release from sympathetic postganglionic nerves and the lack of endogenous adenosine inhibitory tonus. The latter might be explained by the nerve-to-glial redistribution of inhibitory adenosine A_1_ receptors found to occur in hypertensive arteries. These mechanisms would lead to an increase in noradrenaline and ATP release from stimulated sympathetic nerves. Taken this into account one can predict that, in the synaptic cleft, in addition to higher levels of noradrenaline, increased amounts of ATP are likely to accumulate with subsequent vasoconstriction of vascular smooth muscle cells by α_1_ adrenoceptors and P2 receptors activation, respectively. Such changes may have impact in vascular reactivity, contributing to hypertension, renewing the interest of the purinergic system as a target for novel therapeutic strategies.
